# Supplementary material for: Strain-controlled shell morphology on quantum rods
Source: Nat Commun. 2019 Jan 2;10:2. doi: 10.1038/s41467-018-07837-z (PMC6315019; doi:10.1038/s41467-018-07837-z)
Supplement: Supplementary file 1 — Supplementary information [file 41467_2018_7837_MOESM1_ESM.docx]

Supplementary Information for

**Strain-controlled shell morphology on quantum rods**

Botao Ji^1,2^, Yossef E. Panfil^1,2^, Nir Waiskopf^1,2^, Sergei Remennik^2^, Inna Popov^2^, Uri Banin^1,2^*

*Corresponding author. Email: uri.banin@mail.huji.ac.il

^1^Institute of Chemistry, Hebrew University, Jerusalem 91904, Israel. ^2^Center for Nanoscience and Nanotechnology, Hebrew University, Jerusalem 91904, Israel.

**Supplementary Methods**

**1. Finite element simulation of strain in a core/shell nanorod with different morphologies**

Following the works^1,2^, the elastic strain tensor $\varepsilon_{ij}(r)$ defined as:

$${\left( 1.1 \right) \varepsilon}_{ij}\left( r \right)=\frac{1}{2}(\frac{\partial u_{i}}{\partial j}+\frac{\partial u_{j}}{\partial i})$$

where $u\left( r \right)=r-r^{'}$ is the displacement field, $r$ and $r^{'}$ are the location of a point before and after deformation, respectively. $i,j=x,y,z$.

When embedding a material with certain lattice constant inside a different material with different lattice constant, the starting point of the simulation is to make them coherent and hence, to force the embedded material to fit to the shell lattice constant. This creates a strain which exist only in the embedded material.

$${\left( 1.2 \right) \varepsilon}_{ij}^{0}=\frac{a_{i}^{out}-a_{i}^{in}}{a_{i}^{out}}\delta_{ij}$$

where $a_{i}^{out}$and $a_{i}^{in}$ are the lattice constants of the outer material and the embedded material, respectively.

Next, we are letting the core shell structure to relax by deformation $u\left( r \right)$ to the configuration which minimizes the elastic energy. The elastic energy has a quadratic dependence on the strain tensor:

$$\left( 1.3 \right) E=\frac{1}{2}\int\sum_{ijkl} c_{ijkl}\left( r \right)[\varepsilon_{ij}\left( r \right)+\varepsilon_{ii}^{0}\left( r \right)\delta_{ij}]\cdot\left[ \varepsilon_{kl}\left( r \right)+\varepsilon_{kk}^{0}\left( r \right)\delta_{kl} \right]dV$$

where $c_{ijkl}\left( r \right)$ is the elastic modulus tensor. This equation can be viewed as an integration of a functional which depends on $\frac{\partial u_{i}}{\partial i}$ but not on$u\left( r \right)$. So, in order to minimize the integration of this functional we are solving the Euler-Lagrange equation which turns after some algebra to:

$$\left( 1.4 \right) -\sum_{i, k, l=x,y,z} \frac{\partial}{\partial i}\{c_{ijkl}(r)[\frac{\partial u_{k}\left( r \right)}{\partial l}+\varepsilon_{kk}^{0}\left( r \right)\delta_{kl}]\}=0$$

These sets of equations were solved numerically by Comsol Multiphysics software. For the uniqueness of the solution, a fixed core $u=0$was assumed as a constraint to prevent the translation or rotation of the structure. The other outer boundaries were specified as free surfaces due to the zero external force.

Since the corrected strain computed using the shell coordinates, it is needed to be converted to the core coordinates:

$$\left( 1.5 \right) \varepsilon_{ij}\left( r \right)=\frac{1}{2}\left( \frac{a_{i}^{out}}{a_{i}^{in}}\frac{\partial u_{i}}{\partial j}+\frac{a_{i}^{out}}{a_{i}^{in}}\frac{\partial u_{j}}{\partial i} \right)+\frac{a_{i}^{out}}{a_{i}^{in}}\varepsilon_{ii}^{0}\left( r \right)\delta_{ij}$$

The strain energy per particle of the core/shell rod is computed by integration of the strain energy all over the rod volume.

$$\left( 1.6 \right) E_{strain}=\frac{1}{2}\int\sum_{ijkl} c_{ijkl}\left( r \right)\varepsilon_{ij}\left( r \right)\cdot\varepsilon_{kl}\left( r \right)dV$$

The values used for these calculations are summarized in Supplementary Table 1.

**2. Surface energy calculation of a core shell rod with different morphologies**

Following the work^3^, a flat surface core shell rod acts like a wetting layer in which the surface energy has an exponential dependence on the shell thickness $t$ for the transition between the substrate and the film surface energies:

$${\left( 2.1 \right) \gamma}_{WL}(r)=\gamma_{sub}+(\gamma_{film}-\gamma_{sub})\cdot(1-e^{-\frac{r}{h_{0}\eta}})$$

where $\gamma_{sub}$ is the surface energy density of the substrate which was taken as $0.066[\frac{J}{m^{2}}]$. It is worth noting that the values indicated in the literature^4^ for the non-polar faces of ZnSe, are in the range of $0.28-0.49\left[ \frac{J}{m^{2}} \right]$, Nevertheless, these values were calculated for bare ZnSe crystal, However, in chemically synthesized nanocrystals the ligands are reducing the surface energy^5^. $\gamma_{film}$ is the surface energy density of a film with infinite thickness which is assumed to have a uniform crystal structure as the substrate. Since there is lack in the literature about a film of ZnS grown on ZnSe substrate, we assume it to be $\frac{1}{2}\gamma_{sub}$ close to the values reported in the literature^6^. $h_{0}$ is the thickness of one monolayer 0.3 nm. $\eta$ is a dimensionless parameter that depends on the interactions between the layers. We took $\eta$ to be 0.7, like the value reported in the literature^3^.

For different morphologies rather than flat shell, the shell thickness $t'$ varies along the rod, the surface energy density was calculated in the following way. For $t'=t_{WL}$ where $t_{WL}$ is the wetting layer thickness, the surface energy was calculated by Supplementary Equation (2.1). For $t'>t_{WL}$ the surface energy is going back to the substrate surface energy in the following way^3^:

$${\left( 2.2 \right) \gamma}_{oscillating shell}(t')=\gamma_{WL}(t_{WL})+(\gamma_{sub}-\gamma_{WL}(t_{WL}))\cdot(1-e^{-\frac{t'}{h_{0}\eta_{up}}})$$

where $\gamma_{WL}(t_{WL})$ is the surface energy density of the wetting layer with thickness $t_{WL}$ and $\eta_{up}$is 3.5.

The total surface energy per particle was calculated by integration of the surface energy density all over the surface of the rod.

$$\left( 2.3 \right) E_{surface}=\int\gamma_{shell}(r)dS$$

**3. Exploring different morphologies**

As discussed in the main text, different shell morphologies were tested using the general equation:$r\left( \theta,z \right)=r_{flat}\cdot S+c\cdot sin\left( m\theta+\frac{2\pi k}{L_{rod}}z \right)$, where $r\left( \theta,z \right)$ is the distance of the outer shell surface from the rod axis, $r_{flat}$is the shell radius in case of flat morphology, $c$ is the oscillations amplitude of the perturbation in the shell morphology, $m$ is equivalent to the number of islands along the perimeter of the rod, $k$ is equivalent to the number of islands along the rod length and $L_{rod}$ is the rod length. All the morphologies were constrained to have the same shell volume using the parameter $S$:

$$\left( 3.1 \right) S=\sqrt{1-\frac{c^{2}}{2\cdot{r_{flat}}^{2}}}$$

Figure 3k in the main text present the energy difference ($\Delta E)$ between the total surface and strain energy per particle of different shell morphologies compared to the flat shell with the same shell volume corresponding to 4 monolayers shell thickness. For all morphologies the energy difference ($\Delta E)$ presented in the figure is for the oscillations amplitude $c$ which gives the minimum ($\Delta E)$.

| description | symbol | ZnSe | ZnS | Ref. |
| --- | --- | --- | --- | --- |
| Elastic modulus tensor | $c_{xxxx}$ | 10.42·10^10^[Pa] | 12. 2·10^10^[Pa] | ^2,7^ |
| Elastic modulus tensor | $c_{zzzz}$ | 11.65·10^10^[Pa] | 13. 8·10^10^[Pa] | ^2,7^ |
| Elastic modulus tensor | $c_{xxyy}$ | 4.76·10^10^[Pa] | 5. 8·10^10^[Pa] | ^2,7^ |
| Elastic modulus tensor | $c_{xxzz}$ | 3.53·10^10^[Pa] | 4.3·10^10^[Pa] | ^2,7^ |
| Elastic modulus tensor | $c_{xzxz}$ | 2.4·10^10^[Pa] | 2.9·10^10^[Pa] | ^2,7^ |
| Elastic modulus tensor | $c_{xyxy}$ | 2.83·10^10^[Pa] | 3.2·10^10^[Pa] | ^2,7^ |
| Lattice constant $\parallel$ c axis | $a_{\parallel}$ | 6.54·10^-10^[m] | 6.26·10^-10^[m] | ^8,9^ |
| Lattice constant $\perp$ c axis | $a_{\perp}$ | 4.003·10^-10^[m] | 3.82·10^-10^[m] | ^9,10^ |

**Supplementary Table 1**. The summary of the values used for strain energy calculations.

**
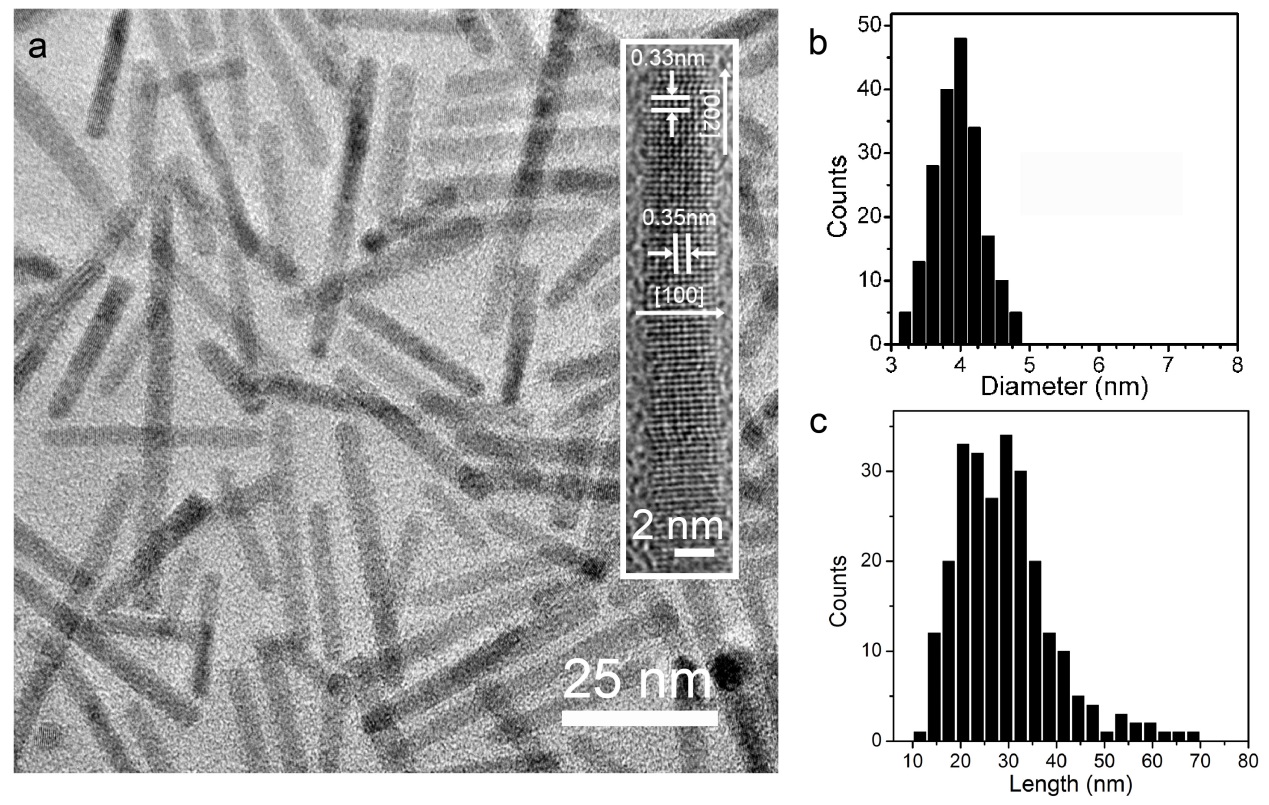
** **Supplementary Figure 1**. (**a**) TEM image of ZnSe nanorods. Inset is the corresponding high-resolution transmission electron microscopy (HRTEM) image. HRTEM measurements on the rods oriented with <010> type zone axis parallel to the incident beam contain two sets of perpendicular lattice fringes with spacing of 0.33 and 0.35 nm corresponding, to (002) and (100) atomic planes, respectively, a typical ZnSe hexagonal wurtzite structure of ZnSe. (**b**) Histogram of diameter of ZnSe nanorods. (**c**) Histogram of length of ZnSe nanorods.


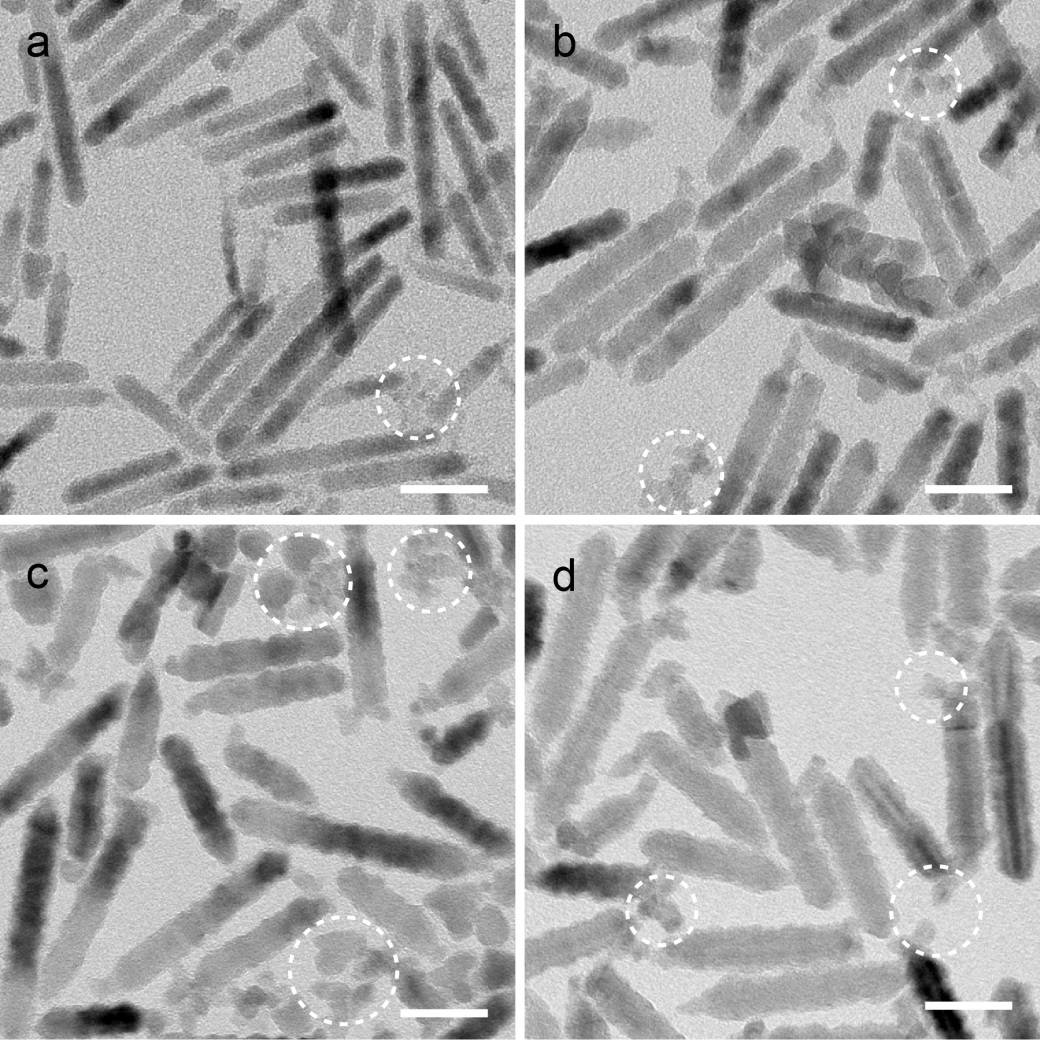


**Supplementary Figure 2**. Shape evolution of ZnSe/ZnS core/flat-shell nanorods in the process of shell growth. Zinc oleate with a 1/4 mole ratio between zinc and oleic acid was used as the shell precursor. (**a-d**) TEM images of ZnSe/ZnS nanorods with increasing thickness of ZnS shell with the reaction time of 60, 120, 180 and 210 min, respectively. Free ZnS NPs due to self-nucleation are marked in white circles in the images. All the scale bars are 25 nm.


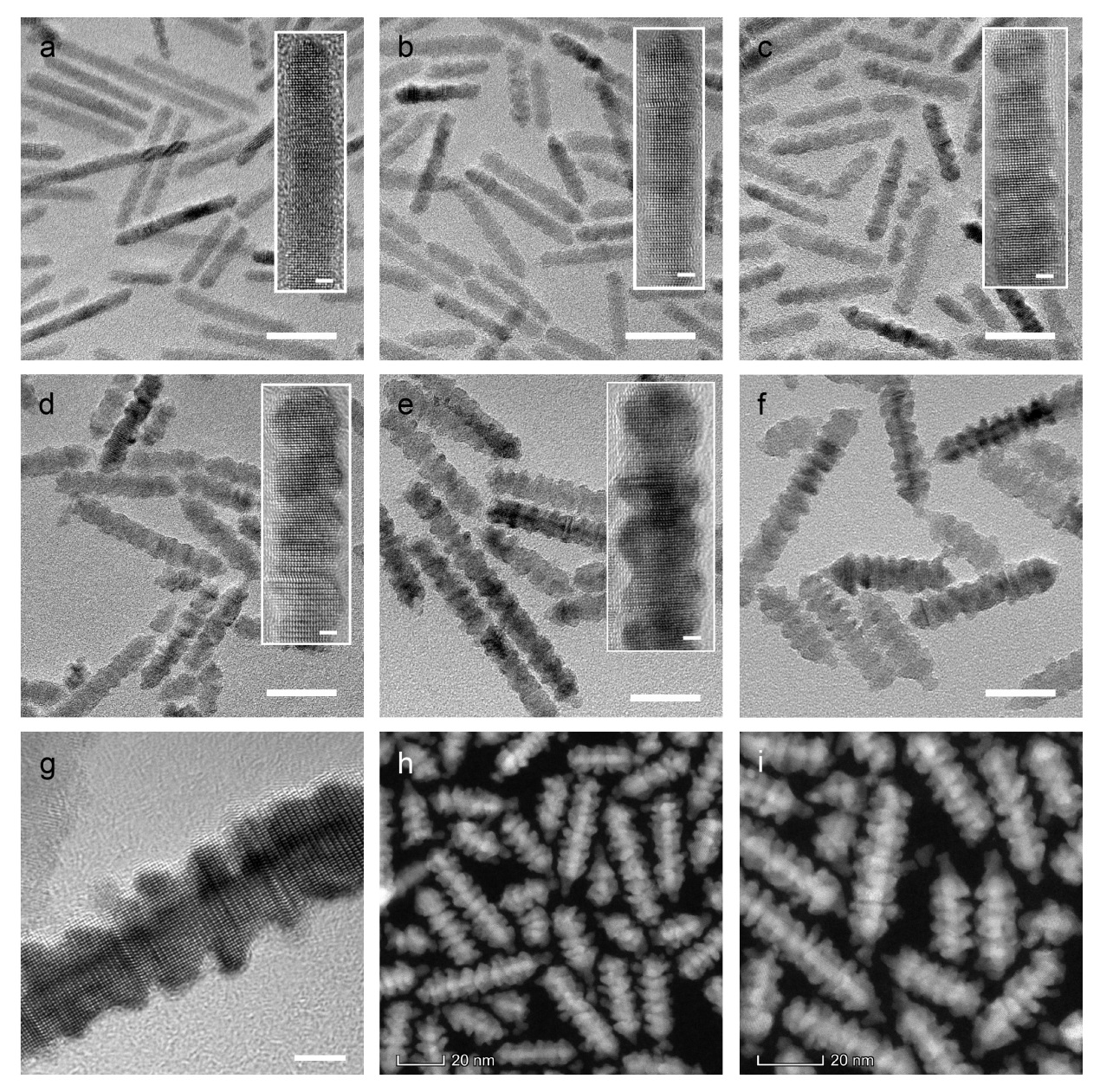


**Supplementary Figure 3**. Shape evolution of ZnSe/ZnS core/islands-shell nanorods in the process of shell growth. Zinc oleate with a 1/6.3 mole ratio between zinc and oleic acid was used as the shell precursor. (**a-f**) TEM images of ZnSe/ZnS nanorods with increasing thickness of ZnS shell with the reaction time of 60, 90, 120, 150, 180 and 210 min, respectively. (**g**) HRTEM image of the sample in (F). (**h, i**) HAADF-STEM images of the sample in (f). Scale bars in (a-f) and (g) are 25 and 5 nm, respectively; the scale bars in insets are 2 nm. Note that short ZnSe nanorods with higher contrast can be recognized also in small nanoparticles as shown in h and i.


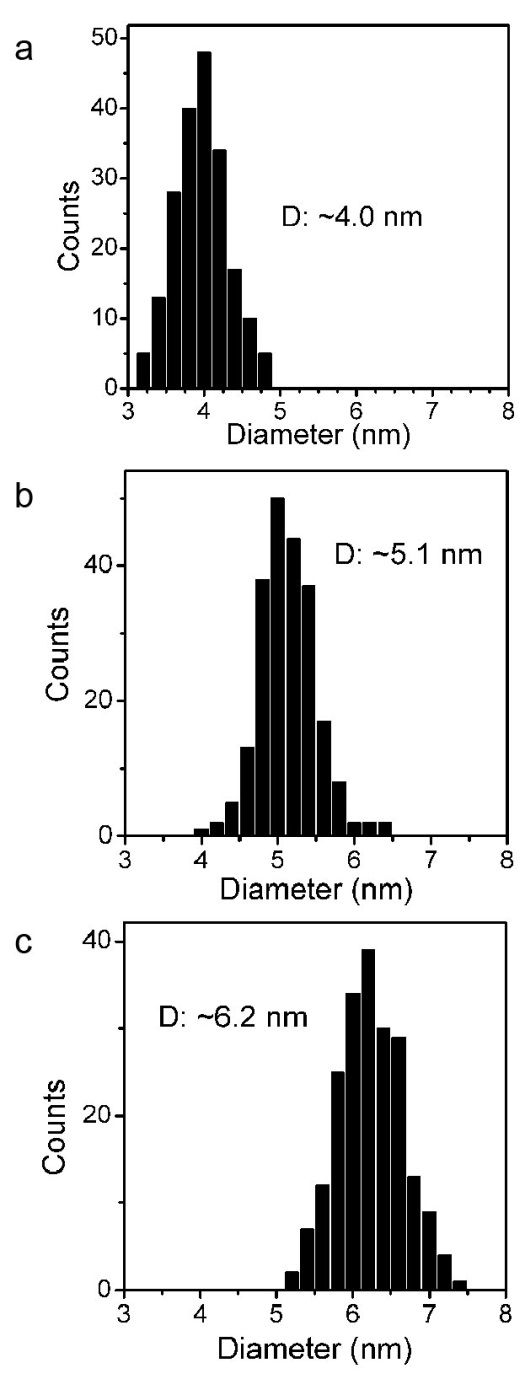


**Supplementary Figure 4**. Histograms of diameters of ZnSe and ZnSe/ZnS core/islands-shell nanorods. (**a**) ZnSe nanorods as shown in Supplementary Figure 1a. (**b-c**) ZnSe/ZnS core/islands-shell nanorods, corresponding to the samples as shown in Supplementary Figure 3a and b, respectively.

**
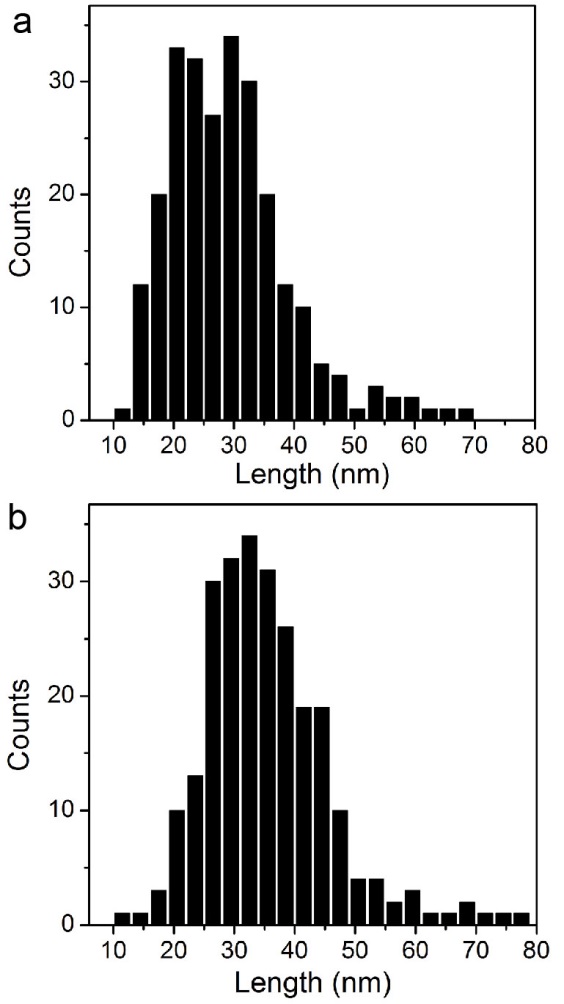
**

**Supplementary Figure 5**. Histograms of length of ZnSe and ZnSe/ZnS core/ shell nanorods. (**a**) ZnSe nanorods as shown in Supplementary Figure 1a. (**b**) ZnSe/ZnS core/ shell nanorods as shown in Supplementary Figure 3b. The length of core/shell nanorods increased a bit upon the shell growth, indicating ripening did not happen in the presence of shell precursors during the shell growth process.

**
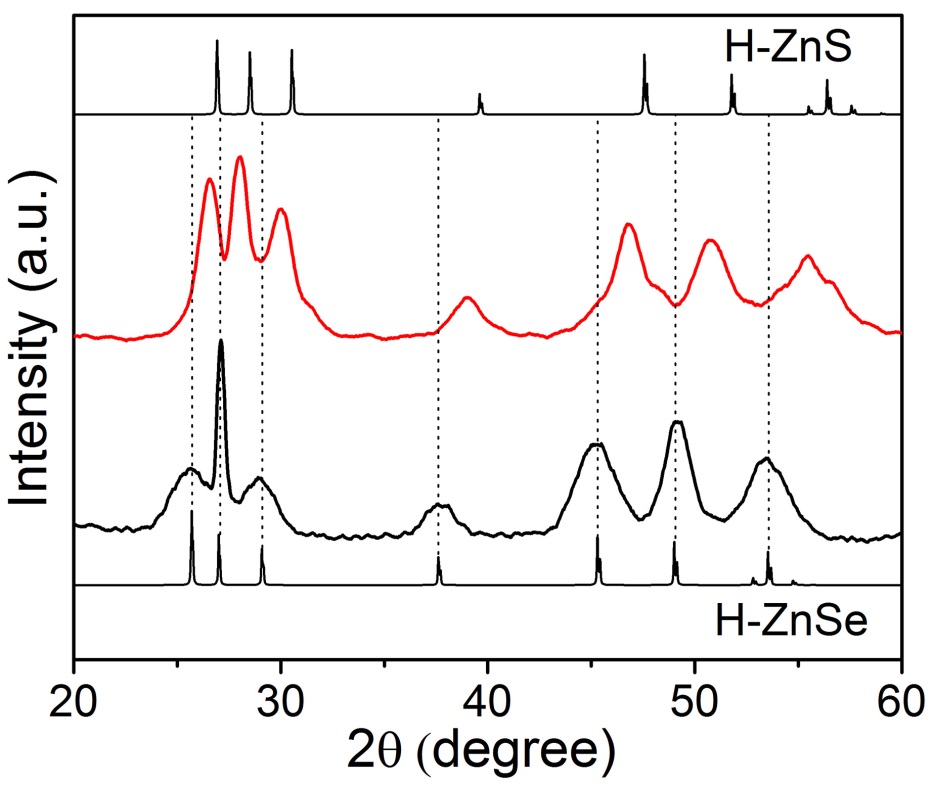
**

**Supplementary Figure 6**. XRD of ZnSe nanorods (black) and ZnSe/ZnS core/islands-shell nanorods (red). The standard XRD patterns of bulk hexagonal ZnSe (JCPDS file no. 00-015-0105) and hexagonal ZnS (JCPDS file no. 00-036-1450) are also shown for comparison. XRD of ZnSe nanorods matches the hexagonal wurtzite ZnSe. The relatively sharper (002) peak at ∼27° indicates the favorable growth along the c-axis. Upon ZnS shell growth, all the diffraction peaks shifted to higher angles, due to the smaller lattice constant for ZnS compared with ZnSe. The original wurtzite crystal structure was maintained, consistent with epitaxial shell formation due to the slow shell deposition.

**
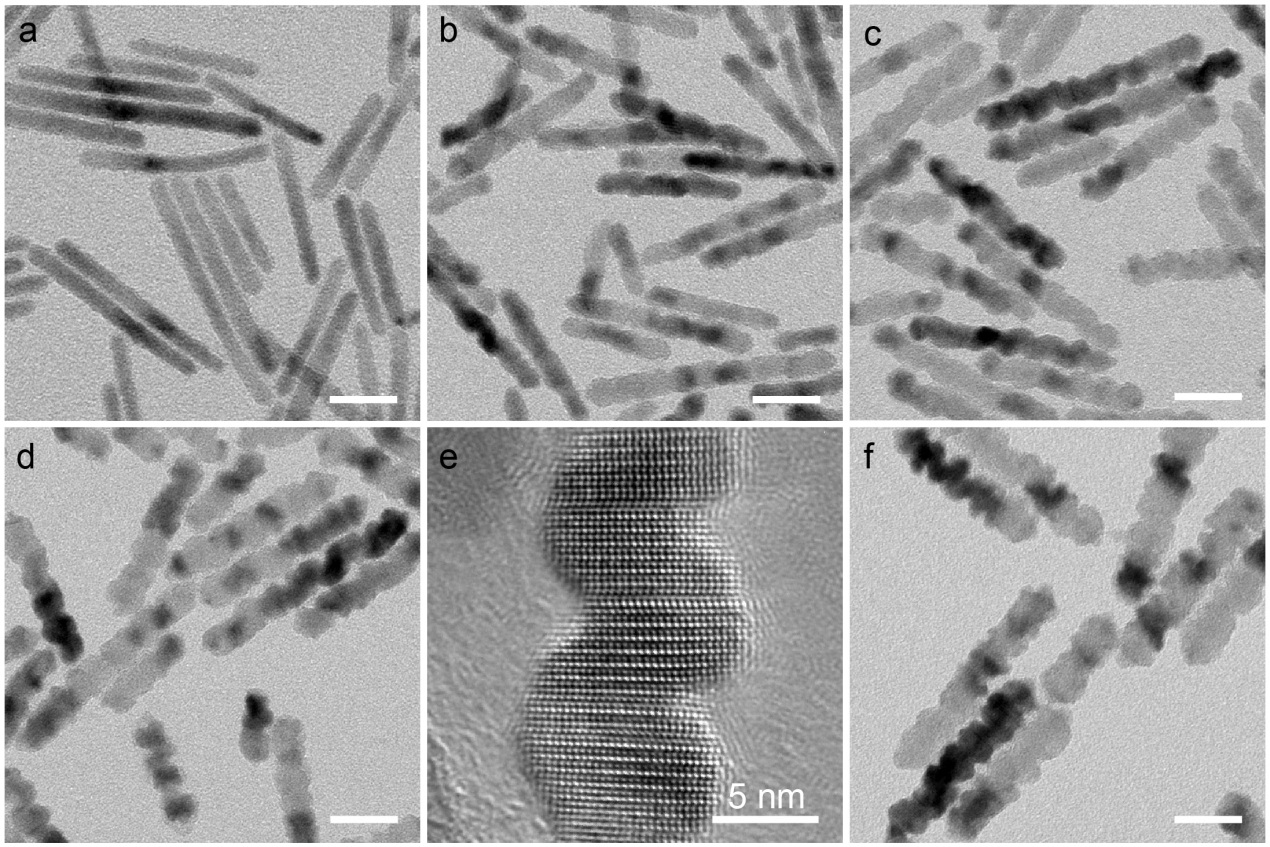
**

**Supplementary Figure 7**. Shape evolution of ZnSe/ZnS core/helical-shell nanorods in the process of shell growth. Zinc oleate with a 1/10 mole ratio between zinc and oleic acid was used as the shell precursor. (**a-d, f**) TEM images of ZnSe/ZnS nanorods with increasing thickness of ZnS shell with the reaction time of 60, 120, 180, 210 and 270 min, respectively. (**e**) HRTEM image of the sample in (d). Scale bars in (a-d) and (f) are 25 nm.


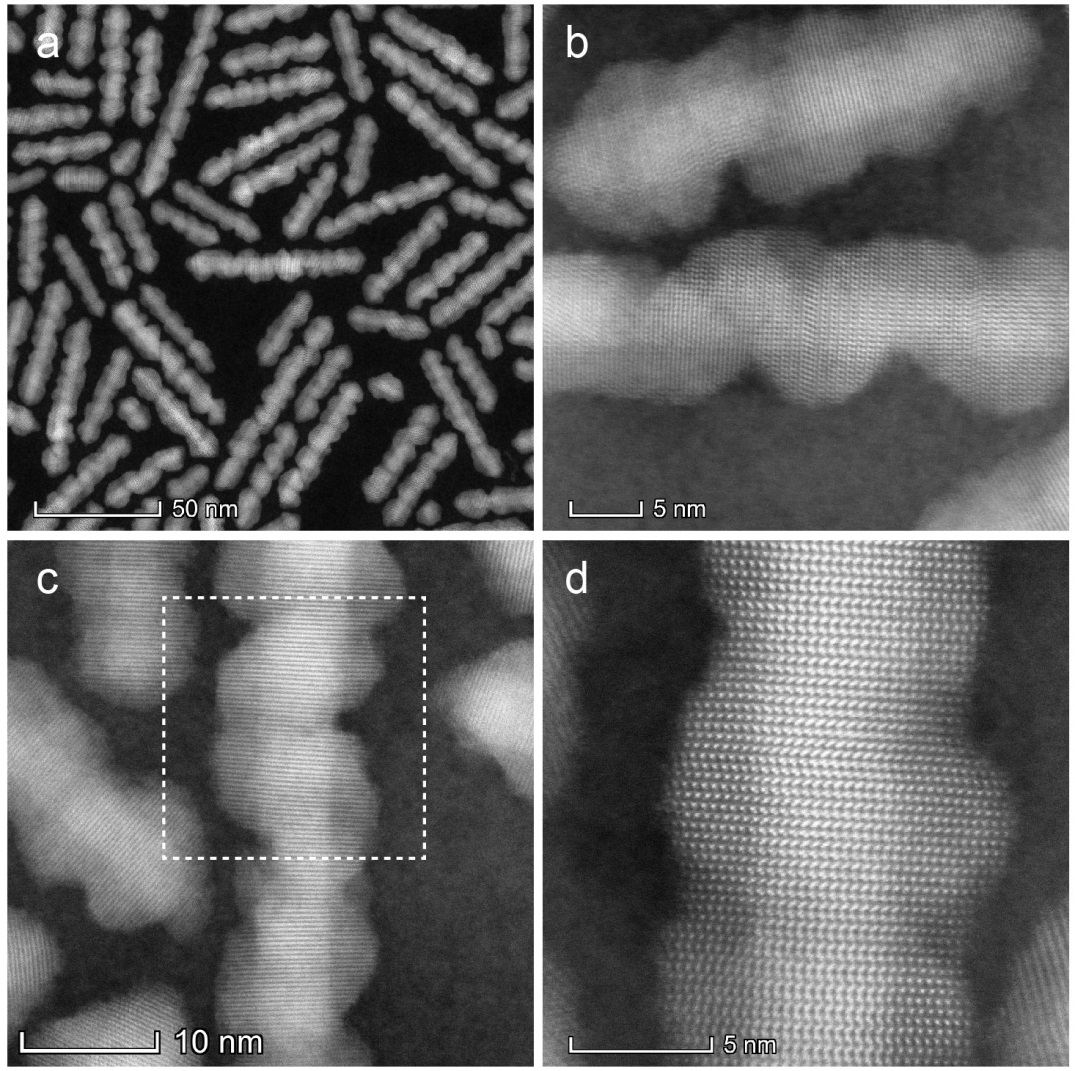


**Supplementary Figure 8**. HAADF-STEM images with different magnifications of ZnSe/ZnS core/helical-shell nanorods (the sample as shown in Supplementary Figure 7d). The dashed square in (c) highlights the site of chirality inversion on one nanorod.

**
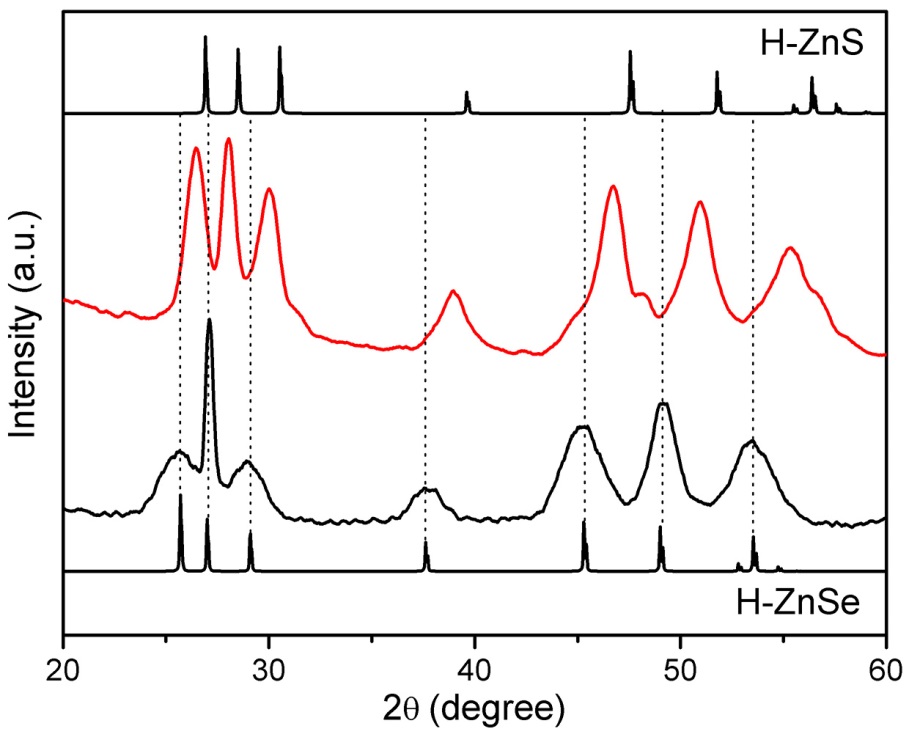
**

**Supplementary Figure 9**. XRD of ZnSe nanorods (black) and ZnSe/ZnS core/helical-shell nanorods (red). The standard XRD patterns of bulk hexagonal ZnSe (JCPDS file no. 00-015-0105) and hexagonal ZnS (JCPDS file no. 00-036-1450) are also shown for comparison.


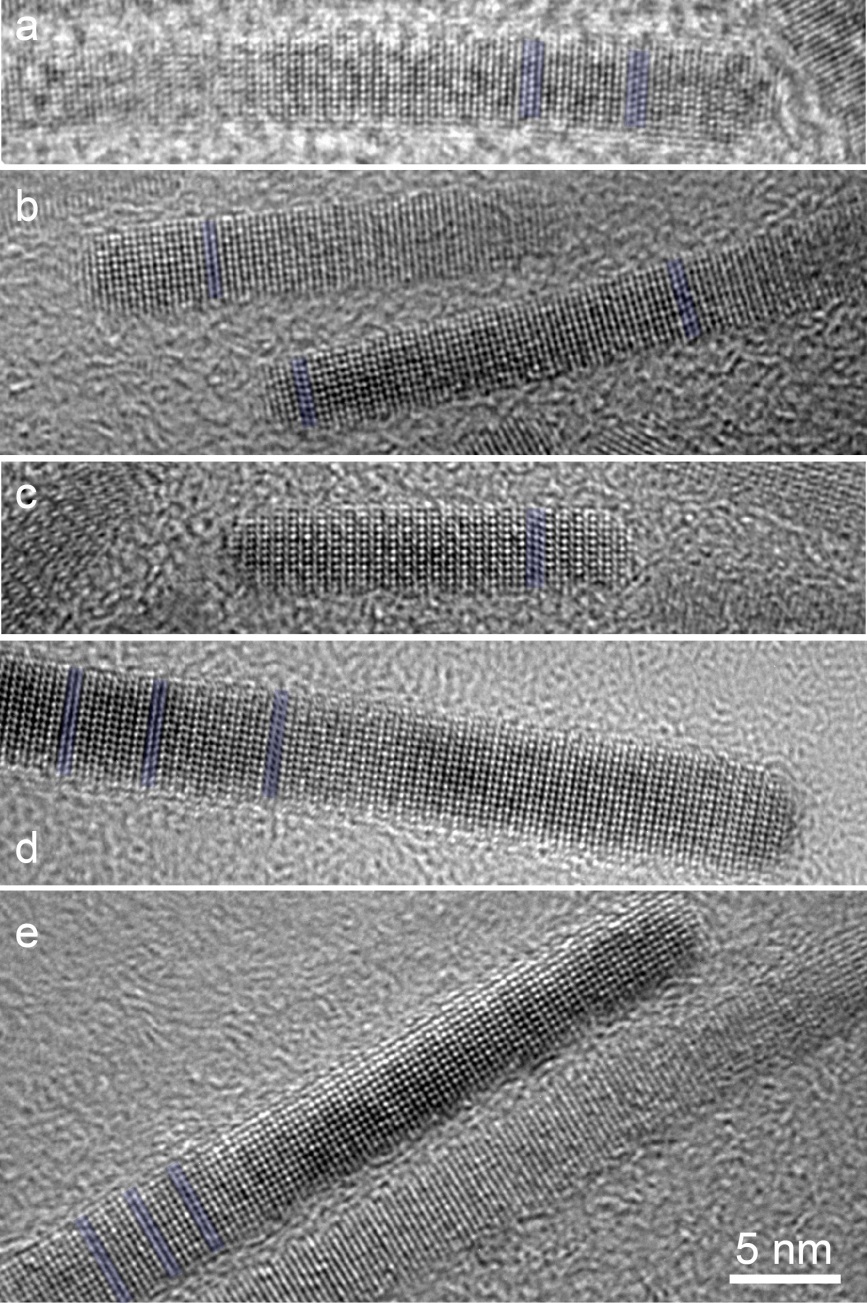


**Supplementary Figure 10**. Stacking faults analyses on the basis of HRTEM images of ZnSe nanorods. Stacking faults are highlighted in blue.


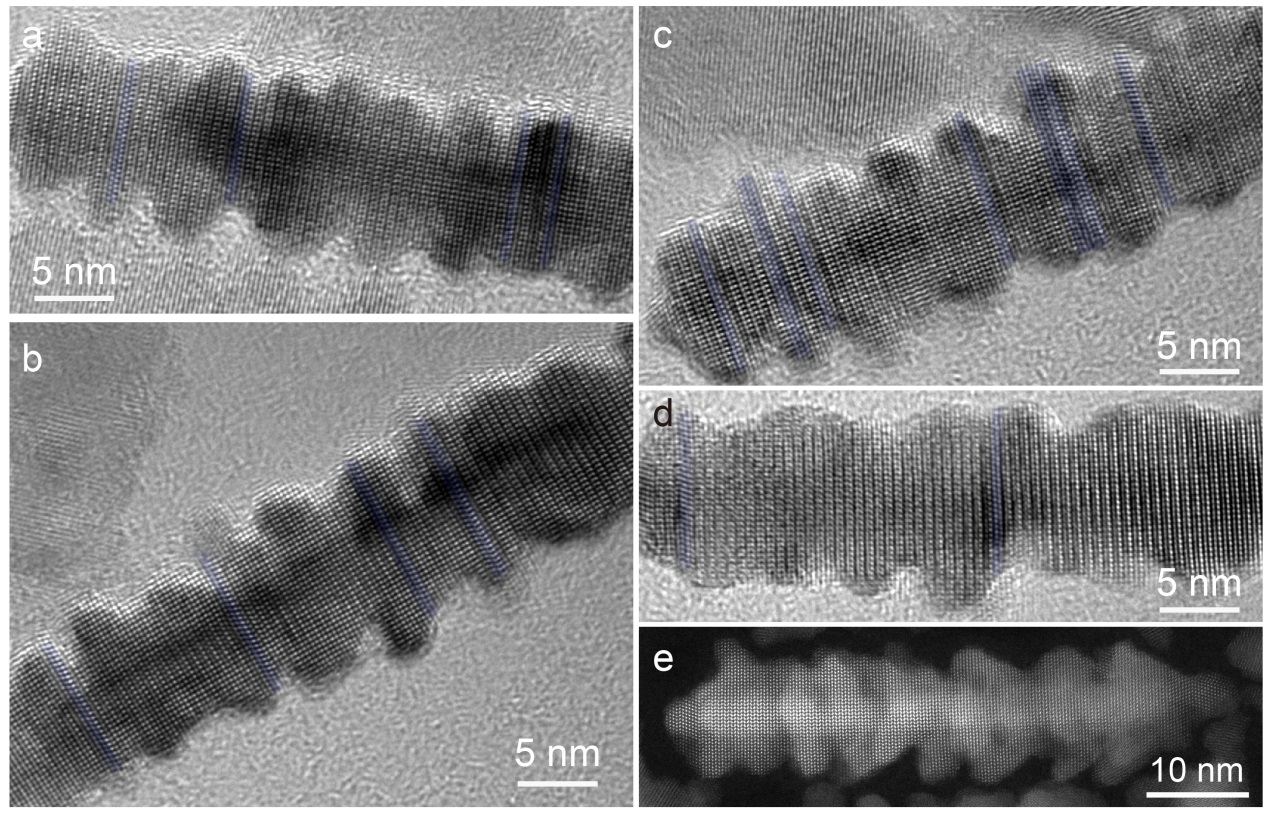


**Supplementary Figure 11**. Stacking faults analyses on the basis of HRTEM images of ZnSe/ZnS core/islands-shell nanorods. Stacking faults are highlighted in blue.

Islands-shell growth may be related to the release of mechanical stress *via* formation of additional stacking faults on the core during the growth of shell. When simultaneous multiple growth sites appear on a nanorod core, the strain due to the lattice mismatch is released not only towards a growing surface of shell , but also towards the core, so that the core could undergo plastic deformation, i.e. structural defects could be induced *via* shuffling the shortest {001} type planes.

The distribution of stacking faults are not uniform in both ZnSe and ZnSe/ZnS nanorods, and the number remains more or less the same or increases a bit after the shell growth (Supplementary Figure 10 and 11). However, the islands density appears relatively uniform along the nanorods. More importantly, the islands-shell growth still happens when there are no stacking faults at all (Supplementary Figure 11e). Thereby, there should be no relation between the initial stacking faults and the morphology of the growing islands-shell.

**
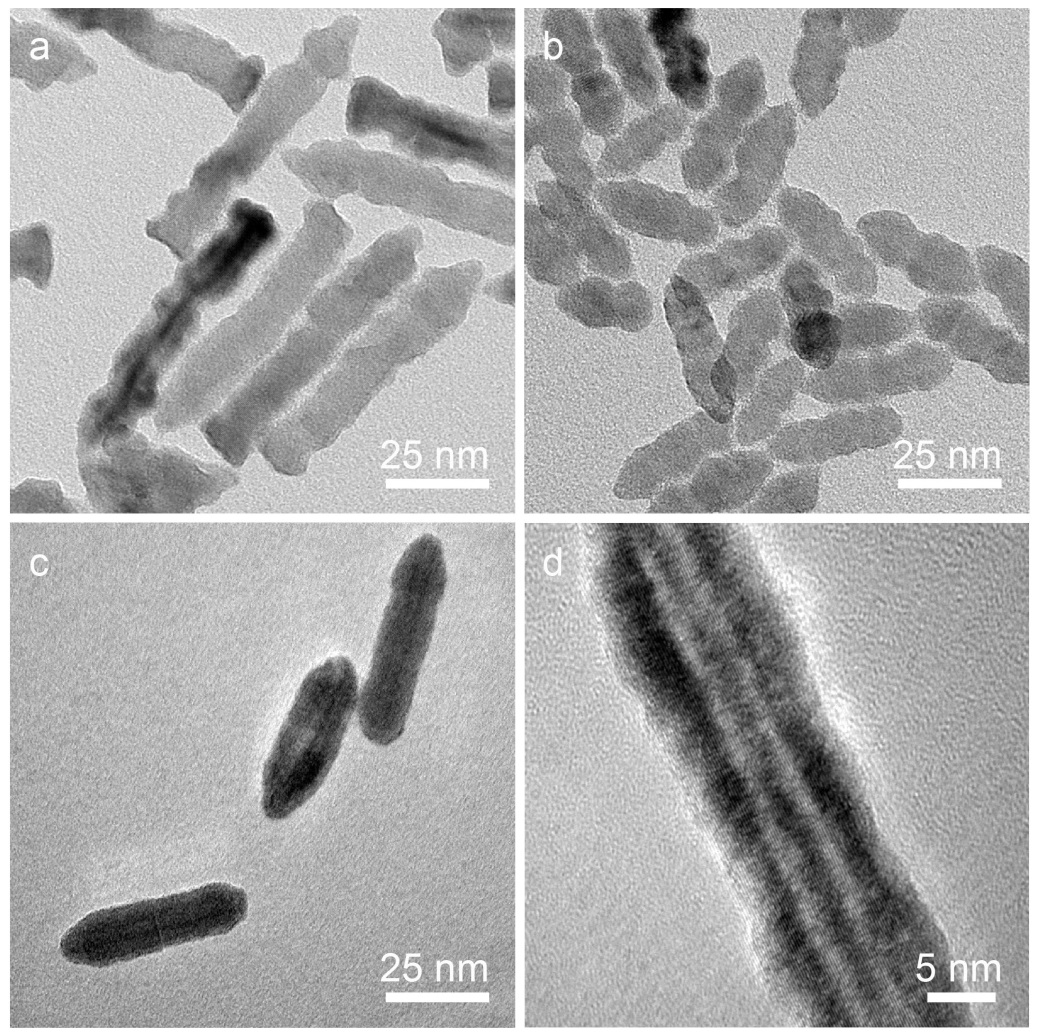
**

**Supplementary Figure 12**. TEM images of (**a**) CdSe nanorods with a thick CdS shell. (**b**) ZnS nanorods with a thick CdS shell. (**c-d**) ZnSe nanorods with a thick CdS shell. In some nanorods, the core/shell structures can be directly visualized.


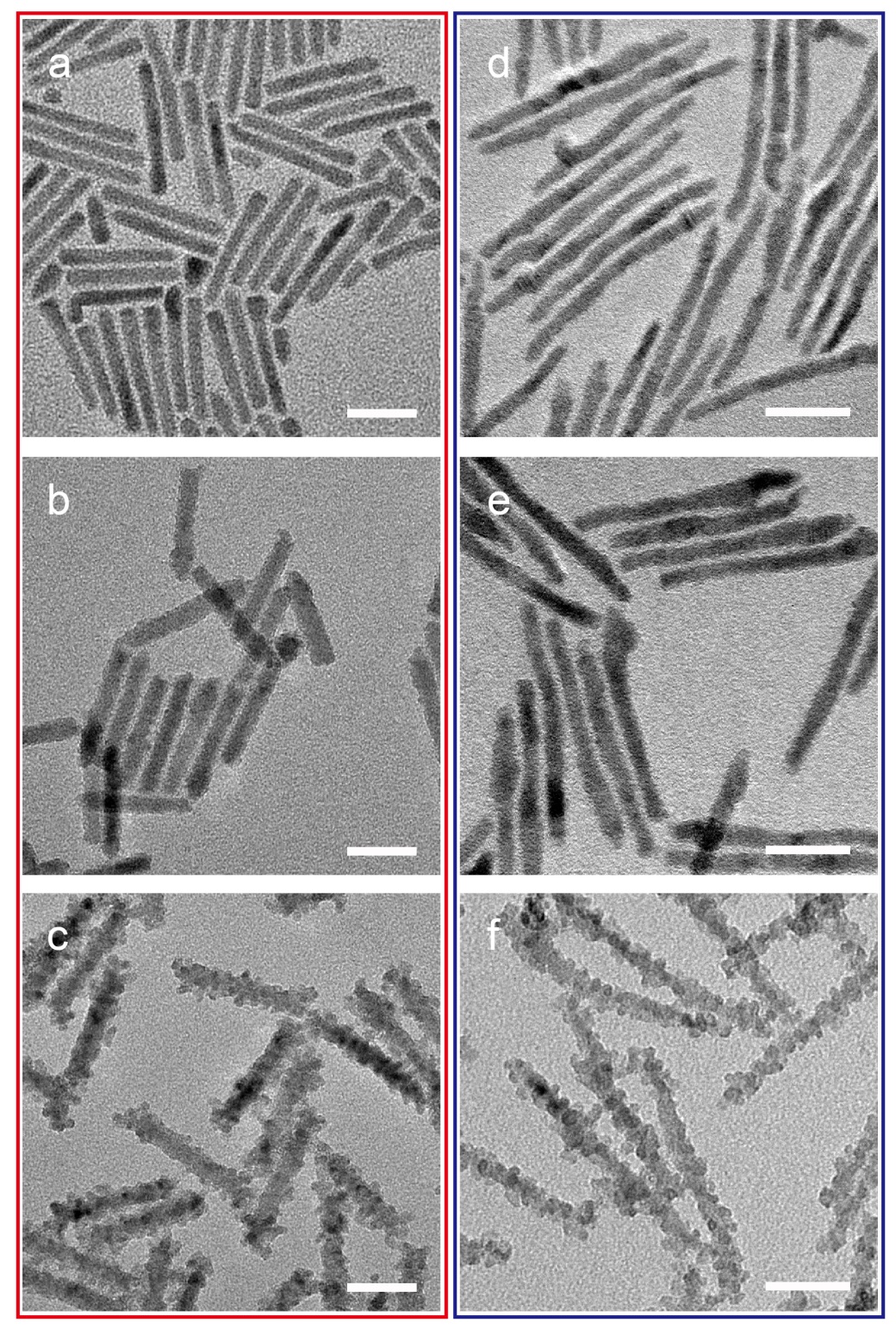


**Supplementary Figure 13**. Shape evolution of ZnS shell growth on CdSe/CdS seeded nanorods (left) and CdSe nanorods (right). (**a**) CdSe/CdS seeded nanorods. (**b**) CdSe/CdS/ZnS core/shell nanorods before the islands growth starts. (**c**) CdSe/CdS/ZnS core/islands-shell nanorods. (**d**) CdSe nanorods. (**e**) CdSe/ZnS core/shell nanorods before the islands growth starts. (**f**) CdSe/ZnS core/islands-shell nanorods. All the scale bars are equal to 25 nm.


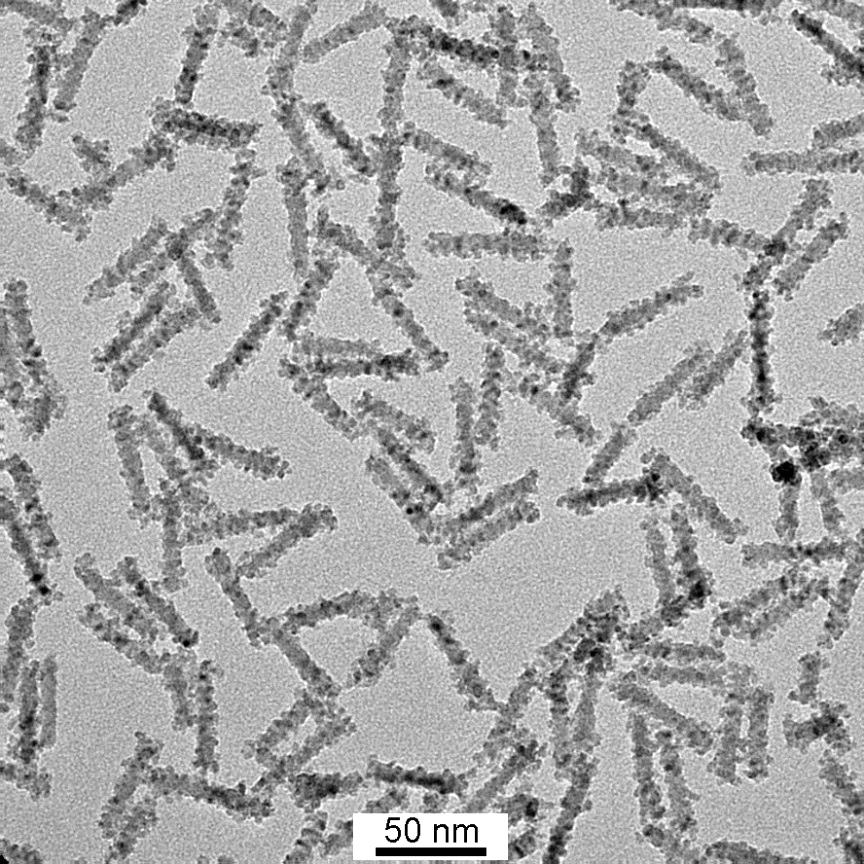


**Supplementary Figure 14**. A large area TEM image of CdSe/CdS/ZnS core/shell nanorods shown in Fig. 2f in the main text.

**
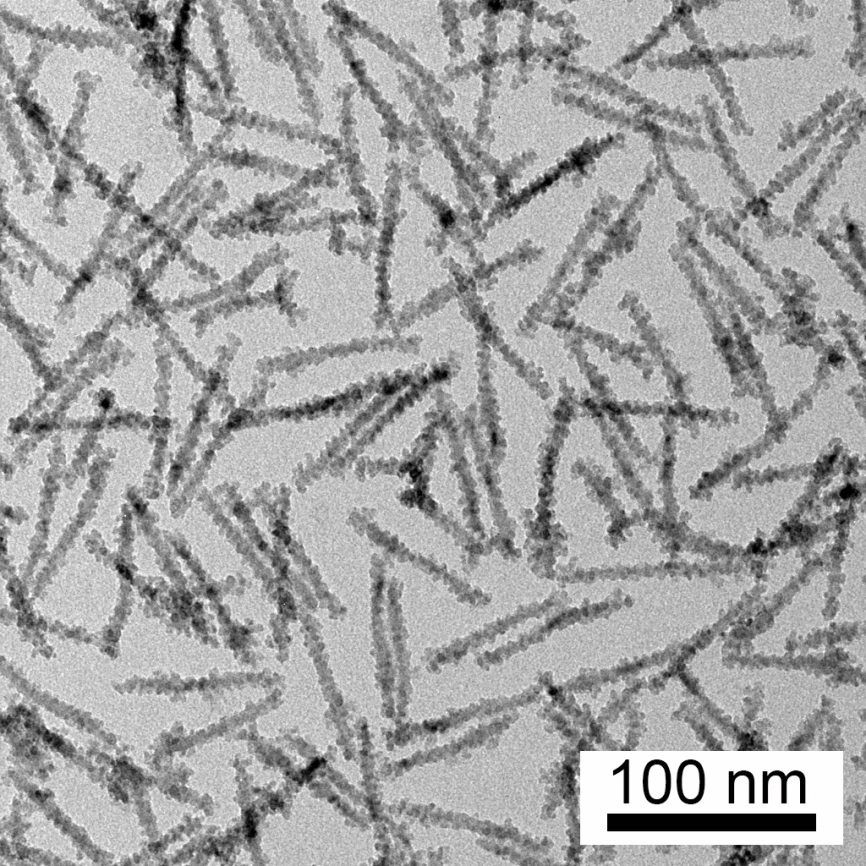
**

**Supplementary Figure 15**. A large area TEM image of CdSe/ZnS core/shell nanorods shown in Fig. 2g in the main text.


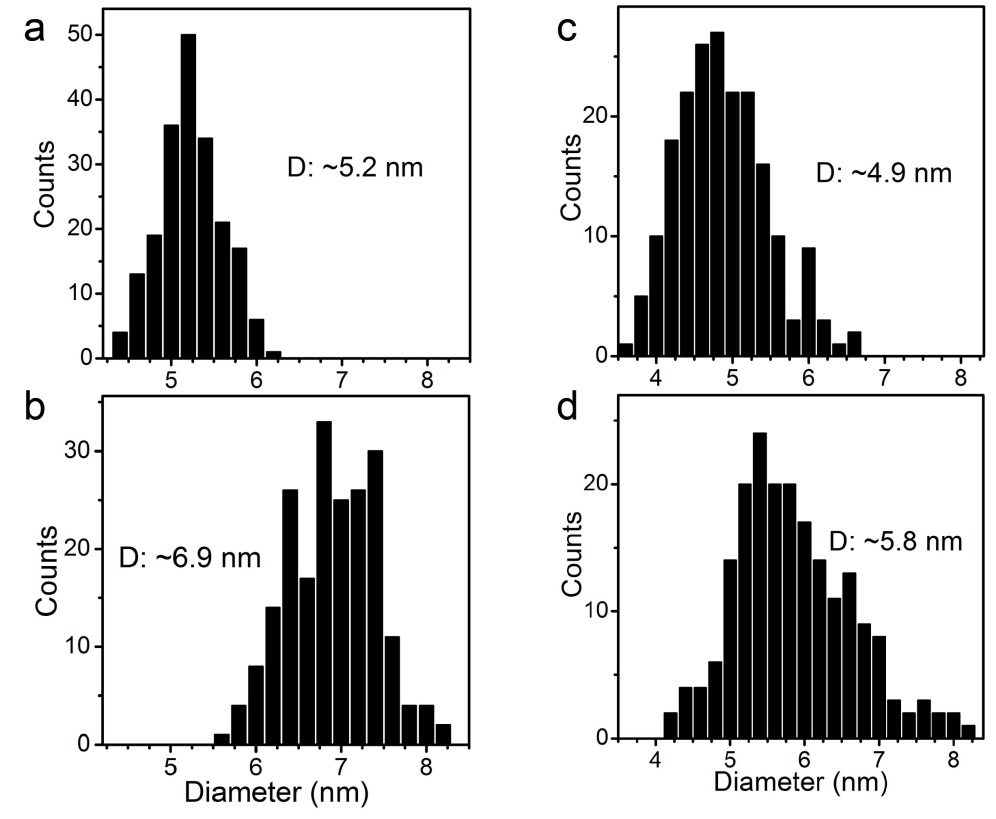


**Supplementary Figure 16.** Histograms of diameter of core/shell nanorods. (**a**) CdSe/CdS seeded nanorods as shown in Supplementary Figure 13a. (**b**) CdSe/CdS/ZnS core/shell nanorods as shown in Supplementary Figure 13b. (**c**) CdSe nanorods as shown in Supplementary Figure 13d. (**d**) CdSe/ZnS core/shell nanorods as shown in Supplementary Figure 13e.


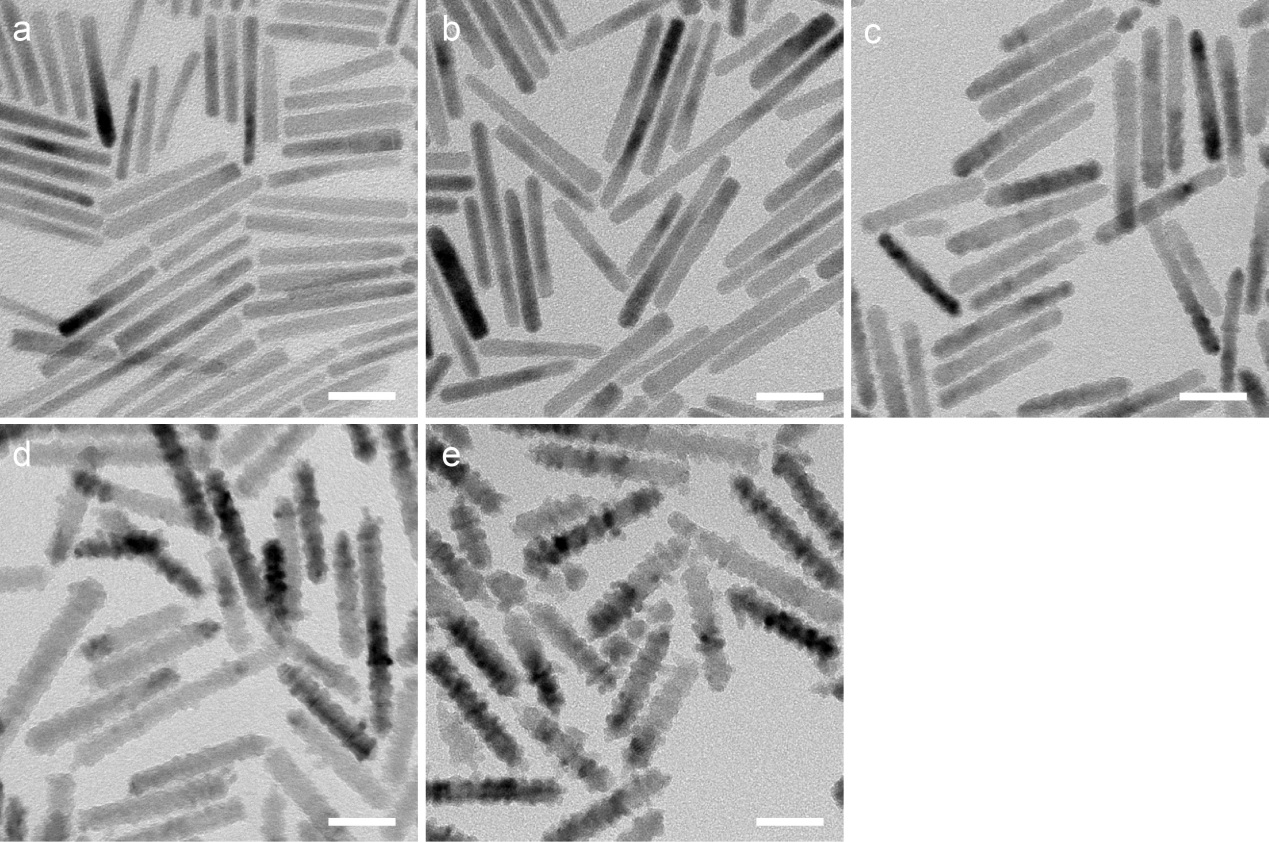


**Supplementary Figure 17**. Evolution of shell morphologies in a typical islands-shell growth of ZnS on ZnSe nanorods with a diameter of 5.5 nm. (**a**) TEM image of ZnSe nanorods. (**b-e**) TEM images of ZnSe/ZnS nanorods with increasing thickness of ZnS shell with the reaction time of 60, 120, 180 and 210 min, respectively. All the scale bars are 25 nm.


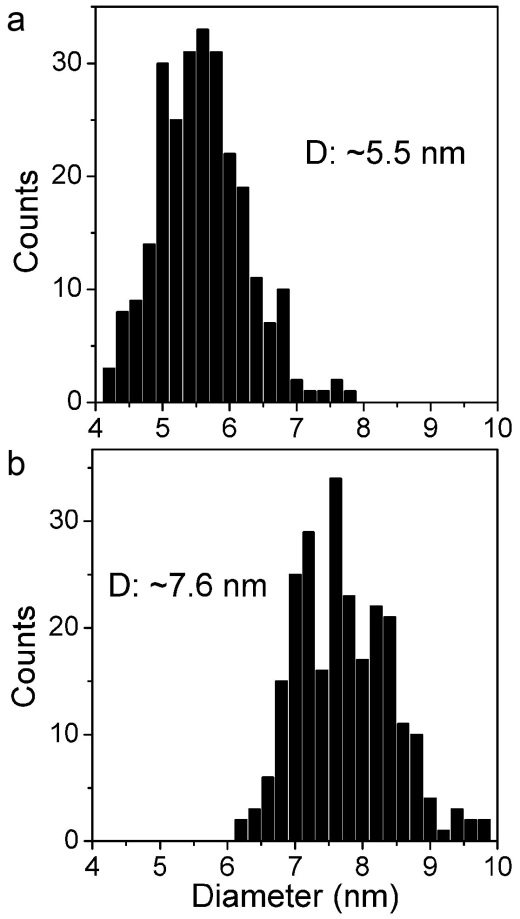


**Supplementary Figure 18.** Histograms of diameter of (**a**) ZnSe nanorods with a diameter of ~5.5 nm and (**b**) ZnSe/ZnS core/shell nanorods as shown in Supplementary Figure 17a and c, respectively.


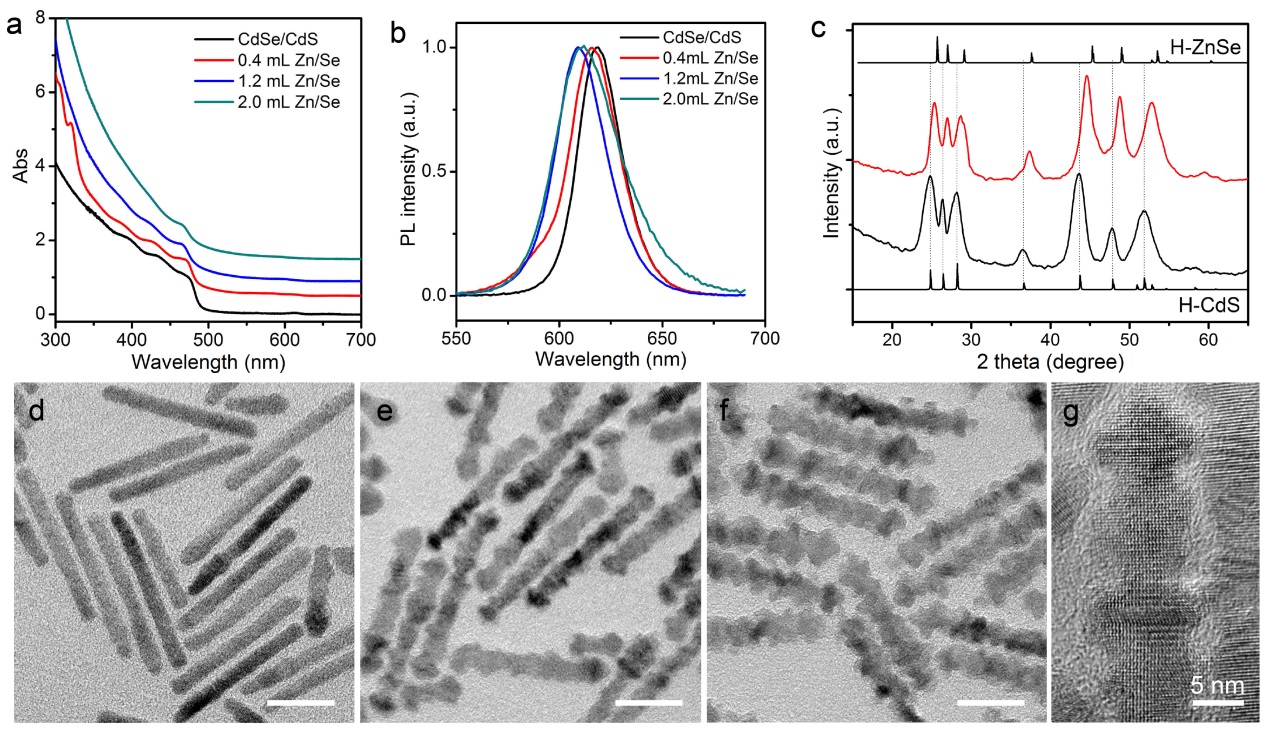


**Supplementary Figure 19**. Evolution of (**a**) absorption and (**b**) emission spectra in the synthesis of ZnSe islands-shell growth on CdSe/CdS seeded nanorods as a function of the volume of ZnSe precursor solutions. (**c**) XRD of CdSe/CdS seeded nanorods (black) and CdSe/CdS/ZnSe core/islands-shell nanorods (red). The standard XRD patterns of bulk hexagonal CdS (JCPDS file no. 00-006-0314) and hexagonal ZnSe (JCPDS file no. 00-015-0105) are also shown for comparison. All XRD diffraction peaks shift to higher angles upon ZnSe growth, during which the core/shell nanorods inherit the hexagonal structure of the core nanorods. (**d-f**) TEM images of CdSe/CdS/ZnSe nanorods after injecting 0.4 mL, 1.2 mL and 2.0 mL of ZnSe precursor solutions, respectively. (**g**) HRTEM image of the sample as shown in (f). The scale bars in (d-f) are 20 nm. Hexagonal ZnSe and CdS have relatively small lattice mismatch (~2.6%). At the synthesis temperature (300^o^C), cation diffusion is suggested^11^. Indeed, the ZnSe shell deposition shifted the excitonic absorption of CdS nanorods to short wavelength, which can be explained by slight decrease of the CdS core diameter due to the cation diffusion. The produced ZnS-CdSe interface provides the necessary lattice strain for islands growth.


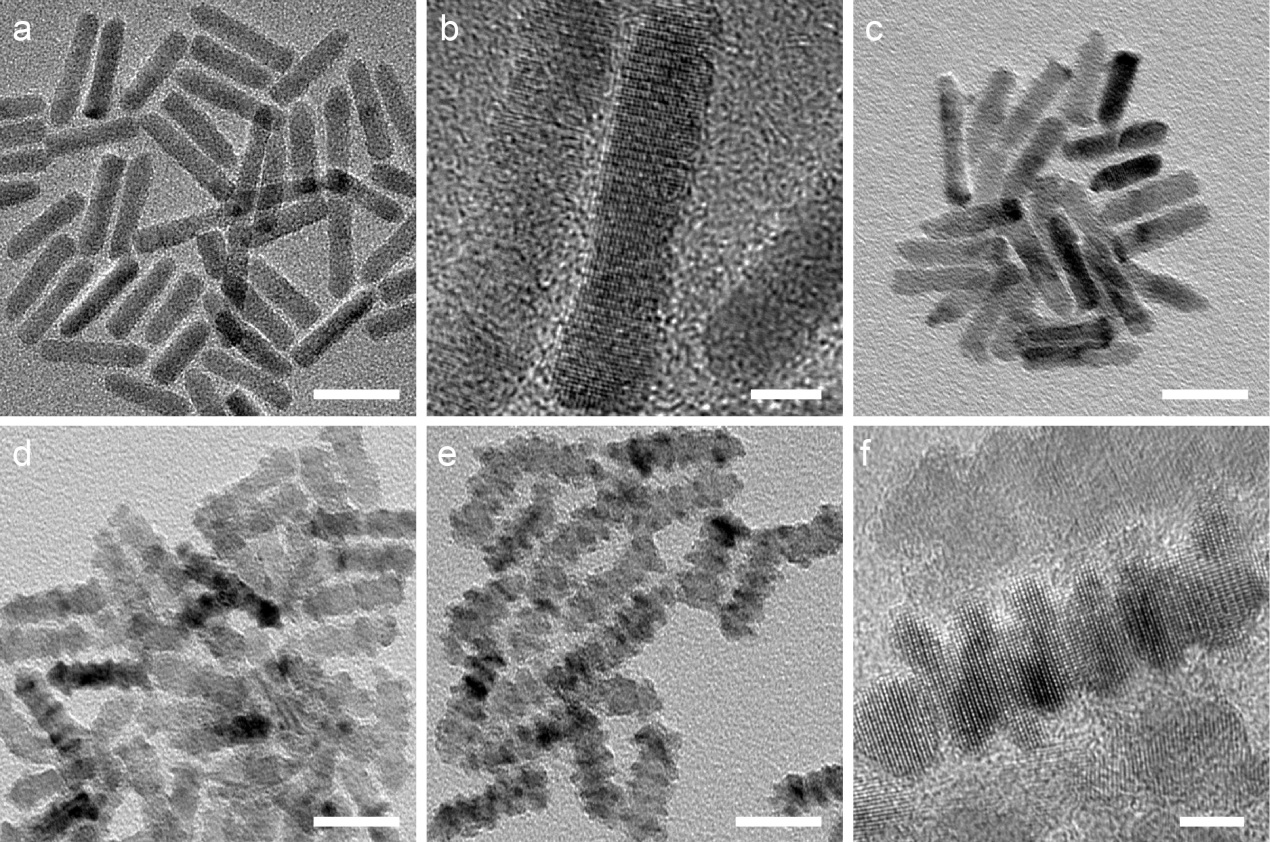


**Supplementary Figure 20**. (**a**) TEM and (**b**) HRTEM images of ZnS nanorods synthesized from CdS nanorods *via* cation exchange reaction. (**c-e**) TEM images of ZnS/ZnSe core/islands-shell nanorods with increasing thickness of ZnS shell with 0.3 mL, 0.6 mL and 1.0 mL of ZnSe precursor solutions being injected, respectively. (**f**) HRTEM image of the sample as shown in (e). Scale bars in (a, c-e) and (b, f) are 25 nm and 5 nm, respectively. The growth of ZnSe on ZnS nanorods first increased the diameter of the nanorods, followed by three-dimensional islands growth, as illustrated by the surface roughness and contrast variation.

**
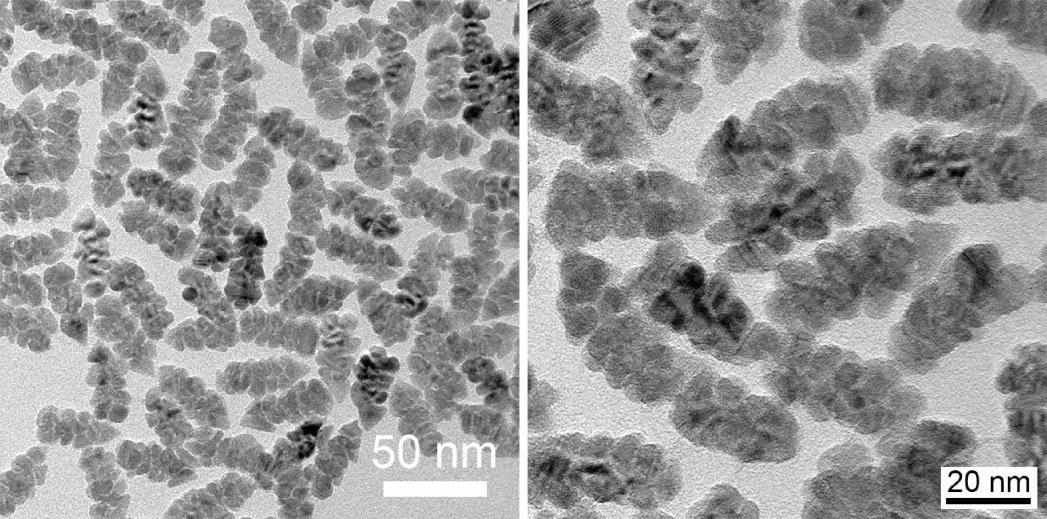
**

**Supplementary Figure 21**. TEM images with different magnifications of ZnS/ZnSe/CdS core/islands-shell/shell nanorods, which were synthesized by growing CdS on ZnS/ZnSe nanorods as shown in Supplementary Figure 20e.


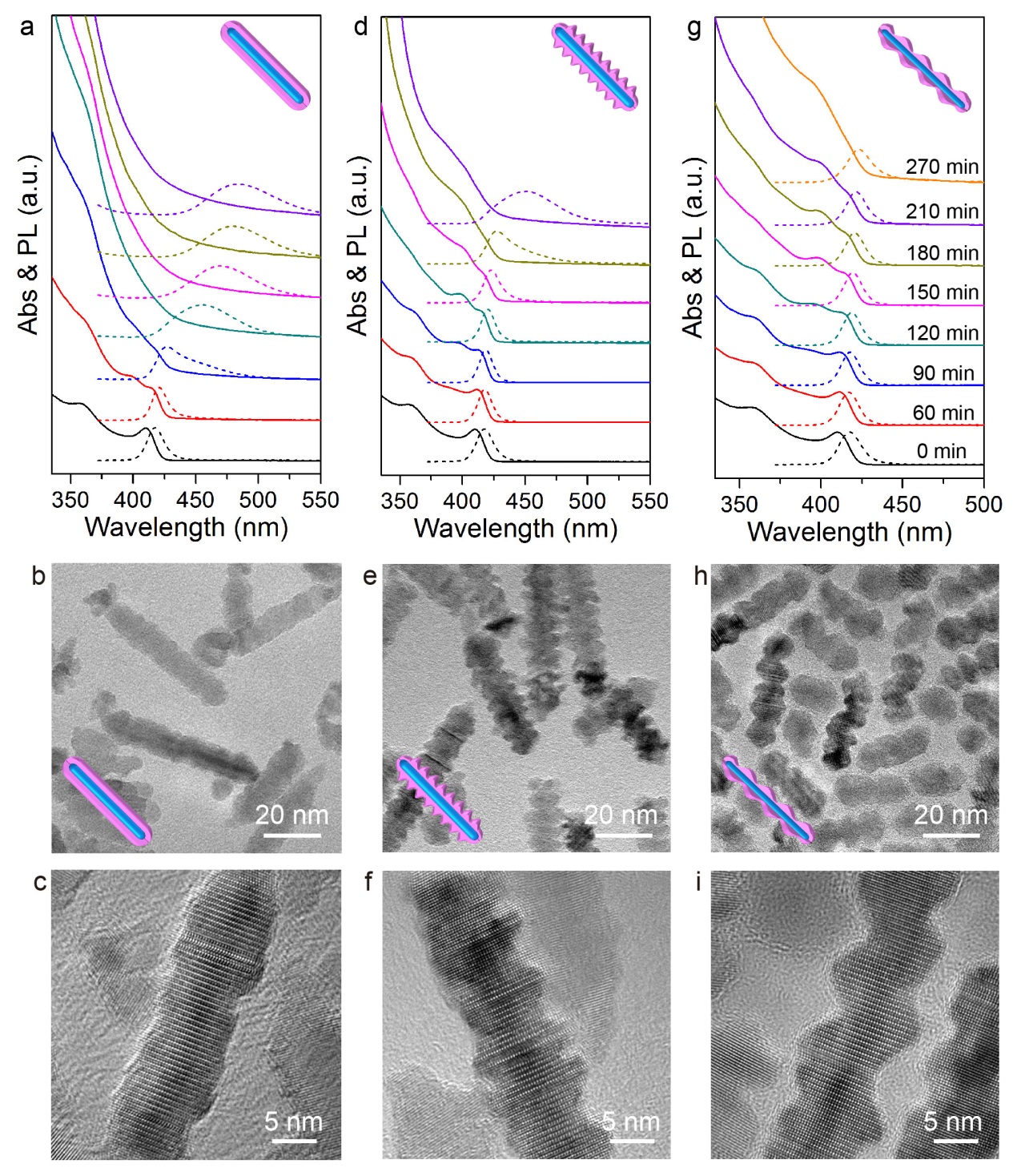


**Supplementary Figure 22**. **Comparison of ZnSe/ZnS core/shell nanorods with different shell morphologies.** (**a-c**) Absorption and emission spectra, TEM and HRTEM images of ZnSe/ZnSe core/flat-shell nanorods, respectively. (**d-f**) Absorption and emission spectra, TEM and HRTEM images of ZnSe/ZnSe core/islands-shell nanorods, respectively. (**g-i**) Absorption and emission spectra, TEM and HRTEM images of ZnSe/ZnSe core/helical-shell nanorods, respectively.


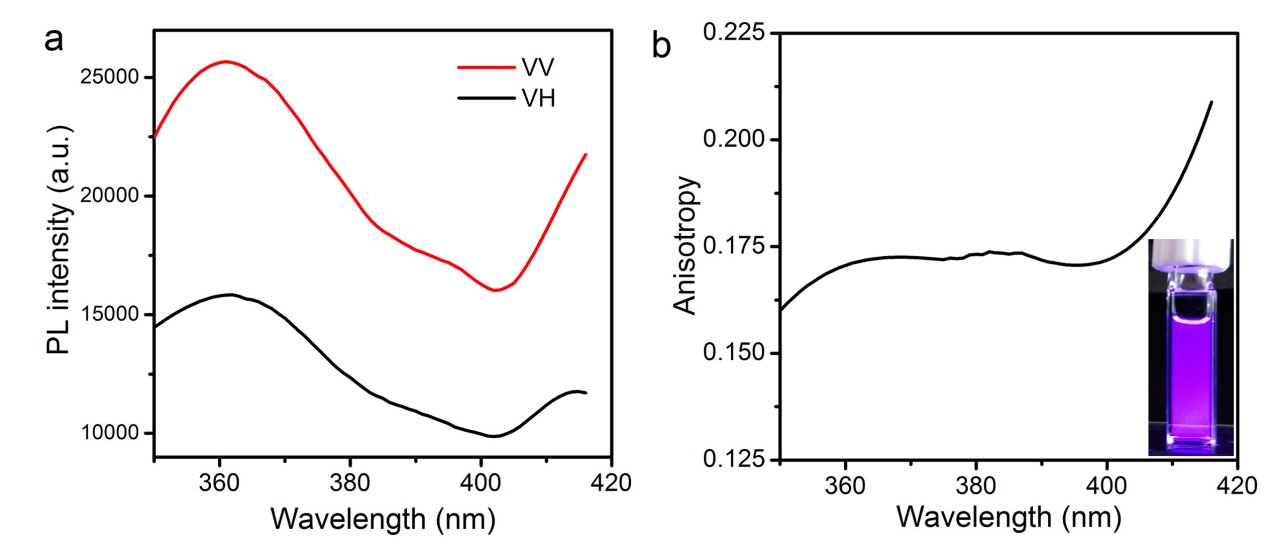


**Supplementary Figure 23**. Fluorescence anisotropy measurements of ZnSe/ZnS core/shell nanorods. **(a**) PLE photo-selection measurements**. (b**) Corresponding fluorescence anisotropy of ZnSe/ZnS nanorods**.** The inset in (b) shows corresponding optical image of ZnSe/ZnS core/shell nanorods (optical density at 360 nm: ~0.1) under UV illumination. The emission polarization of ZnSe/ZnS nanorods is measured by using the excitation photo-selection method. The ZnSe/ZnS nanorods are excited by a vertical light, followed by the measurements of photoluminescence excitation (PLE) spectra parallel (*I_VV_*) and perpendicular (*I_VH_*) to the excitation light. The anisotropy was then extracted according to:

$$r=\frac{I_{VV}-I_{VH}}{I_{VV}+{2I}_{VH}}$$

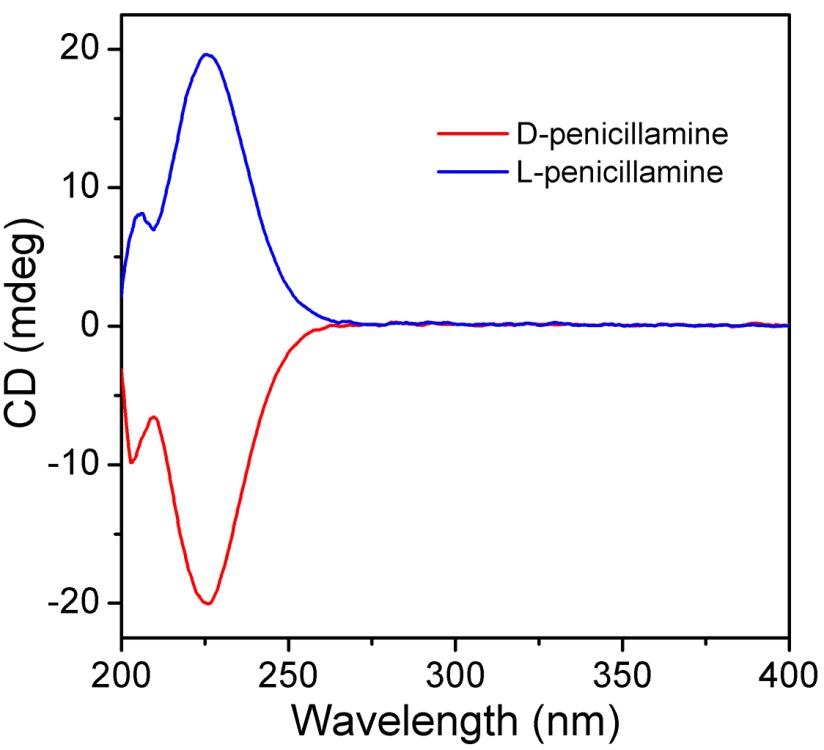


**Supplementary Figure 24**. CD spectra of D- and L-penicillamine aqueous solution.

**Supplementary References**

1. Rajadell, F., Royo, M. & Planelles, J. Strain in free standing CdSe/CdS core-shell nanorods. *J. Appl. Phys.* **111,** 014303 (2012).

2. Yao, Y., Kuroda, T., Dirin, D. N., Sokolikova, M. S. & Vasiliev, R. B. Strain effects on optical properties of tetrapod-shaped CdTe/CdS core-shell nanocrystals. *Superlattices Microstruct.* **76,** 244–252 (2014).

3. Cao, Y. Y., Ouyang, G., Wang, C. X. & Yang, G. W. Physical mechanism of surface roughening of the radial Ge-core/Si-shell nanowire heterostructure and thermodynamic prediction of surface stability of the InAs-core/GaAs-shell nanowire structure. *Nano Lett.* **13,** 436–443 (2013).

4. Jin, L. *et al.* Synthesis and analysis of abnormal wurtzite ZnSe nanowheels. *J. Appl. Phys.* **102,** 044302 (2007).

5. Gong, K. & Kelley, D. F. A predictive model of shell morphology in CdSe/CdS core/shell quantum dots. *J. Chem. Phys.* **141,** 194704 (2014).

6. Li, X. L. & Yang, G. W. Theoretical determination of contact angle in quantum dot self-assembly. *Appl. Phys. Lett.* **92,** 171902 (2008).

7. Mayanovic, R. A., Sladek, R. J. & Debska, U. Elastic constants of Zn_1-x_Mn_x_Se: Tetrahedral bond weakening due to Mn 3d(t2)aaSe 4p hybridization. *Phys. Rev. B* **38,** 1311–1315 (1988).

8. Martin, R. M. Relation between elastic tensors of wurtzite and zinc-blende structure materials. *Phys. Rev. B* **6,** 4546–4553 (1972).

9. Park, Y. S. & Chan, F. L. Photoconductivity spectral response and lattice parameters of hexagonal ZnSe. *J. Appl. Phys.* **36,** 800–801 (1965).

10. Schröer, P., Krüger, P. & Pollmann, J. First-principles calculation of the electronic structure of the wurtzite semiconductors ZnO and ZnS. *Phys. Rev. B* **47,** 6971–6980 (1993).

11. Zhong, X., Han, M., Dong, Z., White, T. J. & Knoll, W. Composition-tunable Zn_x_Cd_1-x_Se nanocrystals with high luminescence and stability. *J. Am. Chem. Soc.* **125,** 8589–8594 (2003).
